# Electrospun Molecularly Imprinted Polymers for Environmental Remediation: A Mini Review

**DOI:** 10.3390/polym17152082

**Published:** 2025-07-30

**Authors:** Sisonke Sigonya, Bakang Mo Mothudi, Olayemi J. Fakayode, Teboho C. Mokhena, Paul Mayer, Thabang H. Mokhothu, Talent R. Makhanya, Katekani Shingange

**Affiliations:** 1 Department of Physics, School of Science, Engineering and Technology, University of South Africa, Pretoria 0002, South Africafakayaj@unisa.ac.za (O.J.F.); 2 DSTI/Mintek NIC, Advanced Materials Division, Mintek, 200 Malibongwe Drive, Randburg 2194, South Africa; tebohom@mintek.co.za; 3 Department of Chemistry and Biomolecular Sciences, University of Ottawa, 150 Louis-Pasteur Pvt, Ottawa, ON K1N 6N5, Canada; paulmichael.mayer@uottawa.ca; 4 School of Chemical and Physical Sciences, University of Mpumalanga, Mbombela 1200, South Africa; 5 Department of Chemistry, Durban University of Technology, Durban 4001, South Africa; talentm@dut.ac.za; 6 Department of Physics, University of the Witwatersrand, Braamfontein 2050, South Africa; katekani.shingange@wits.ac.za

**Keywords:** non-steroidal anti-inflammatory drugs, antiretroviral drugs, electrospinning, molecularly imprinted membranes, environmental remediation, pollution removal

## Abstract

This review critically examines the recent advancements in the development and application of electrospun molecularly imprinted polymer (MIP) nanofiber membranes for environmental remediation. Emphasizing the significance of these materials, the discussion highlights the mechanisms by which electrospun MIPs achieve high selectivity and efficiency in removing various pollutants, including dyes, heavy metals, and pharmaceutical residues such as NSAIDs and antiretroviral drugs. The synthesis methodologies are explored in detail, focusing on the choice of monomers, templates, and polymerization conditions that influence the structural and functional properties of the membranes. Characterization techniques used to assess morphology, surface area, porosity, and imprinting efficacy are also examined, providing insights into how these parameters affect adsorption performance. Furthermore, the review evaluates the performance metrics of electrospun MIPs, including adsorption capacities, selectivity, reusability, and stability in complex environmental matrices. Practical considerations, such as scalability, regeneration, and long-term operational stability, are discussed to assess their potential for real-world applications. The article concludes with an outline of future research directions, emphasizing the need for multi-template imprinting, integration with existing treatment technologies, and field-scale validation to address current limitations.

## 1. Introduction

Pharmaceuticals have been a growing concern as water contaminants, posing threats to both human health and the environment. Non-steroidal anti-inflammatory drugs (NSAIDs) and antiretrovirals (ARVs) have received special attention because of their extensive usage and durability in aquatic settings. Conventional water treatment methods frequently fail to properly remove these substances, forcing the search for novel treatments [1,2]. The use of molecularly imprinted polymers (MIPs), which have emerged as a potential technique for targeted pollutants removal, is one such option [3,4]. Because of their unique structural properties and specific binding capabilities, use of electrospun MIPs has gained recognition in recent years as an effective method of removing water contaminants, such as dyes and heavy metals [5].

Pharmaceuticals, such as NSAIDs and ARVs, are widely utilized for a variety of medicinal purposes and are regularly found in wastewater treatment plant effluents and natural water sources. Their presence in bodies of water is caused by insufficient metabolism and excretion by people and animals, as well as poor disposal techniques [6,7]. These substances can infiltrate the environment via sewage systems and eventually end up in rivers, lakes, and groundwater, posing significant dangers to aquatic life and even human populations that rely on these water supplies. As a result, new technologies that can specifically target and eliminate pharmaceutical contaminants from water sources are crucial.

MIPs have emerged as a viable approach for the selective removal of water contaminants such as pharmaceuticals. MIPs are man-made materials with particular recognition sites for target molecules [8,9,10]. They are made via a technique known as molecular imprinting, in which a template molecule guides the development of corresponding binding holes within the polymer matrix. After removing the template molecule, the resultant MIP preserves the imprinted cavities that can detect and attach to the target molecule [11,12]. These printed cavities are characterized by their chemical functionalities, specific size, and three-dimensional shape, which enable them to recognize and rebind the target molecule with high specificity and strong affinity. The cavities are typically created during the polymerization process in the presence of a template, using monomers that possess functional groups complementary to the target. After polymerization, the template is removed through washing steps or, depending on the MIP format, solid-phase synthesis methods can be used to immobilize the template onto a surface [9]. Beyond their use in removing pollutants from water, MIPs have found diverse applications. These include chromatographic separation [13,14], capture of radioactive waste [15], drug delivery [8,16], recognition of peptides, biomolecules, and cells [17], use as sensors [18,19] and solid-state extraction [20,21,22].

Because of their complicated chemical structures and low quantities, pharmaceuticals are difficult to remove from water. Traditional water treatment procedures, including coagulation, flocculation, and activated carbon filtration, are typically ineffective at totally removing these contaminants [23]. Electrospun MIPs have received a lot of attention in recent years as a viable way to remove contaminants from water. The technology of electrospinning is used to create ultrafine fibers with dimensions ranging from nanometers to micrometers [24]. Electrospinning, when paired with molecular imprinting, allows for the creation of MIPs with increased surface area, porosity, and mechanical characteristics. The resultant electrospun MIPs have a great degree of versatility, allowing them to be integrated into a variety of water treatment systems.

The electrospun MIPs’ unique structural properties, such as their high surface area-to-volume ratio and porous nature, allow for better interaction between the target pollutants and the selective binding sites within the MIP matrix. This improves the membranes’ adsorption capacity and efficiency, enabling for the effective removal of water contaminants such as dyes, heavy metals, and possibly NSAIDs and ARVs [3,25,26,27]. Several studies have demonstrated the effectiveness of electrospun MIPs in removing water pollutants, particularly pharmaceuticals [9,28]. These membranes have shown high adsorption capacities, excellent chemical stability, and good reusability, making them a promising solution for water treatment applications. The use of electrospun MIPs provides a sustainable and efficient approach to selectively remove pollutants from water sources, mitigating potential risks associated with pharmaceutical contamination [9,25,26].

The prevalence of pharmaceuticals as water contaminants, such as NSAIDs and ARVs, provides a substantial challenge to water treatment methods. The advent of MIPs, on the other hand, presents a potential alternative for targeted pollution removal. Electrospun MIPs have shown great promise because of their distinct structural properties and specific binding capabilities. Further research and development in this area can help to promote effective and sustainable water treatment methods, protecting both human health and the environment. Therefore, this review provides an overview of current successful technologies for the removal of pharmaceutiscal pollutants and further proposes electrospun molecularly imprinted membranes as a viable solution for the removal of emerging pollutants in the future.

### 1.1. Molecularly Imprinted Polymers

MIPs have proven to be an enthralling field of study and technology, changing molecular recognition and opening new possibilities for a wide range of applications [9,14,18,19,29]. These custom-made synthetic materials are intended to detect and bind target molecules with high specificity, like the natural lock-and-key process seen in biological systems. As a result, MIPs have several benefits over standard identification systems, making their study an intriguing field of research [30]. One of MIPs’ significant qualities is their adaptability and broad application. These polymers may be made to bind to a wide range of target molecules, such as tiny chemical compounds, peptides, proteins, and even large biomacromolecules [31,32,33]. Because of their capacity to imitate biological recognition processes, researchers can develop selective and robust MIPs for a variety of applications, including environmental monitoring, pharmaceutical delivery sensors, catalysis, and separation science [9,13,14,17,19].

The synthesis of MIPs includes a method known as molecular imprinting, which uses a template-driven approach to produce particular binding sites within the polymer matrix. Researchers can develop MIPs with particular recognition capabilities by carefully choosing the template molecule, monomers, crosslinkers, and polymerization conditions. Because of its tunability, very precise binding sites may be created, ensuring accurate target identification even in complicated sample matrices. MIPs’ durability and dependability are two of their most significant features. MIPs, unlike natural receptors or antibodies, do not denature, degrade, or lose function in severe environments [12]. They are resistant to a broad variety of temperatures, pH levels, and organic solvents, making them excellent for applications requiring robust and long-lasting molecular recognition.

MIPs have clear promise in a variety of industries. MIP-based sensors provide quick and selective detection of contaminants, such as heavy metals, pesticides, and pharmaceutical residues in environmental monitoring [25,31]. See the review by Yang et al. [34] for a more in-depth examination of the components of MIMs, synthesis techniques, surface modification, characterization, performance assessment, and mechanism studies.

In the context of environmental remediation, MIPs serve as tailor-made recognition elements capable of selectively capturing target pollutants from complex matrices [35,36]. By embedding imprinted binding sites directly within a polymeric network, MIPs mimic the high specificity of biological receptors yet withstand harsh chemical and thermal conditions [37]. This robustness makes them particularly attractive for removing persistent organic contaminants, such as phenolic compounds and pharmaceuticals, from wastewater streams [38]. In electrospun nanofiber applications, MIPs bring molecular selectivity to high-surface-area membranes, enabling rapid adsorption kinetics and low detection limits in flow-through systems.

Integration of MIPs with electrospun fibers uses the nanofibrous morphology to maximize accessible binding sites and facilitate mass transport [39]. In a typical “in situ” approach, pre-polymerization mixtures containing template molecules, functional monomers, crosslinkers, and initiators are electrospun directly, yielding fibers whose internal microdomains are imprinted upon subsequent template removal [40]. Alternatively, “coating” methods deposit thin MIP layers onto pre-formed electrospun scaffolds via surface polymerization or grafting [41]. Both strategies produce composites with fiber diameters in the 100–500 nm range, combining the high porosity of nanofibers with the molecular recognition of MIPs to achieve rapid uptake and high capacity for trace pollutants [42].

Recent studies demonstrate that electrospun MIP membranes can achieve adsorption capacities exceeding 50 mg g^−1^ for phenolic endocrine disruptors and sub-ppb selectivity in mixed organic solvent–water matrices [42]. For example, nanofibrous conductive sensor for Limonene reached equilibrium within 200 s under continuous flow, outperforming bulk MIPs by a factor of three in kinetics [43]. In another work, dual-template imprinting enabled simultaneous extraction of atrazine and simazine from agricultural runoff, with recovery rates above 90% after five regeneration cycles [44]. These results highlight the capability of electrospun MIPs to address multi-analyte remediation scenarios while maintaining reusability.

Fabrication parameters critically influence the performance of electrospun MIPs. The choice of functional monomer (e.g., methacrylic acid, acrylamide), crosslinker content, and template concentration determines the affinity and density of binding sites [45]. Solvent system and polymer concentration affect fiber morphology, whereas applied voltage, tip-to-collector distance, and flow rate affect fiber uniformity and porosity [46,47]. Optimizing these variables is essential for balancing high binding capacity with structural integrity; for instance, increasing crosslinker ratios enhances site stability but may reduce porosity, whereas higher monomer concentrations improve imprint fidelity at the expense of fiber fineness.

Despite promising results, challenges remain before widespread deployment of electrospun MIP membranes in large-scale environmental applications. Green synthesis approaches, such as using deep eutectic solvents or biodegradable polymers, are needed to minimize ecological footprint [48]. Scale-up of electrospinning processes requires novel collector designs and multi-nozzle systems to achieve commercial throughput. Furthermore, long-term performance under real wastewater conditions, potential fouling issues, and cost–benefit analyses against existing treatment technologies must be rigorously evaluated. Addressing these gaps will pave the way for robust, selective, and sustainable remediation platforms based on electrospun molecularly imprinted polymers.

### 1.2. Electrospinning

**Figure 1:**
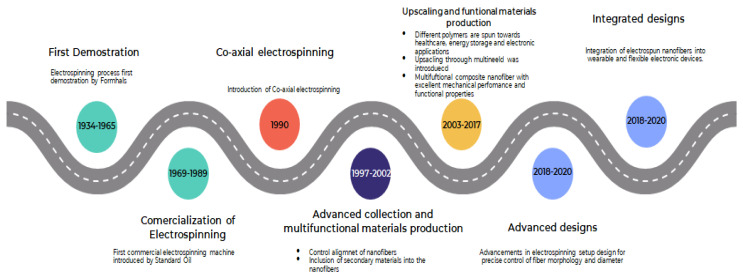
Electrospinning advancement over the years.

Electrospinning, a versatile technique for fabricating ultrafine fibers, has gained significant attention in various scientific fields due to its ability to produce nanofibers with unique properties [49]. Electrospinning has grown and varied over the years, with applications in tissue engineering, filtration, sensors, and energy storage, as shown in Figure 1 [50,51].

In the year 2002 the first electrospun nanofibers were successfully manufactured, ushering in a new age of materials engineering with previously unheard-of characteristics and uses [52].

Further electrospinning processes were developed in 2006, permitting the manufacturing of nanofibers with regulated diameters and better mechanical characteristics. Some key factors that have influenced the manufacturing of nanofibers with regulated diameters and improved mechanical characteristics in electrospinning include polymer concentration, solvent type, and solution viscosity; all these have an impact on the spinning process and the resulting nanofiber shape, including diameter control. The shape and alignment of the nanofibers may be influenced by optimizing the electrospinning setup, which includes the collector design, distance between the needle and collector, and applied voltage. This breakthrough cleared the door for a variety of industrial uses, such as filtration, tissue engineering, and energy storage [53].

In 2010, researchers made significant advances by combining diverse functional elements into electrospun nanofibers, allowing applications such as medication delivery, sensors, and flexible electronics [54,55]. In 2022 researchers devised scalable and continuous electrospinning technologies, allowing for large-scale manufacture of commercial nanofibers [24]. This breakthrough pushed electrospinning closer to industrial usage, with possible applications in the filtration, aerospace, and energy industries. Figure 1 further shows a timeline of important breakthroughs and milestones in the field of electrospinning over the years. The goal of this roadmap is to present a complete picture of the tremendous progress made in this dynamic and multidisciplinary topic.

The incorporation of MIPs into electrospinning is a particularly interesting breakthrough that has significantly broadened the possibilities of both technologies and enabled greater molecular recognition. Electrospinning has various benefits that make it an excellent platform for MIP incorporation. For starters, the approach enables the fabrication of large surface area nanofibers with adjustable shape, such as porous architectures or aligned orientations [56]. This property is critical for MIPs because it improves binding site accessibility and promotes efficient molecular recognition. Researchers increased molecule recognition selectivity and sensitivity by combining MIPs with electrospun nanofibers. Prior to electrospinning, MIPs engineered to selectively bind to certain target molecules can be added into the polymer solution, resulting in MIP-infused nanofibers. The composite materials that arise have improved molecular recognition capabilities, allowing for highly selective detection, separation, and capture of target analytes [9]. No single procedure is entirely liable for all these outcomes. Instead, advances in electrospinning equipment design, polymer selection, solution parameters, and process optimization have all contributed to greater control over nanofiber diameter management and improved mechanical qualities.

The following are some significant elements that have influenced the production of nanofibers with controlled diameters and better mechanical properties in electrospinning: Polymer concentration, solvent type, and solution viscosity can all have an impact on the spinning process and the resulting nanofiber shape, including diameter control. The formation and alignment of the nanofibers may be influenced by optimizing the electrospinning setup, which includes the collector design, distance between the needle and collector, and applied voltage. Choosing suitable polymers with desirable features, such as high molecular weight, excellent solubility, and mechanical strength, might help achieve improved mechanical properties in the resultant nanofibers. Post-treatment techniques, such as annealing, crosslinking, and surface changes, can improve the mechanical characteristics of electrospun nanofibers even more.

The combination of electrospinning with MIPs has resulted in advances in a variety of industries. Electrospun MIP-based nanofibers have been used in biosensors to detect biomarkers, poisons, and pathogens with high sensitivity and selectivity [27]. These nanofiber-based sensors have rapid response times and may be readily incorporated into portable or wearable devices for on-site monitoring.

Electrospun MIPs have shown considerable promise in drug delivery systems in addition to sensing. MIPs can selectively release pharmaceuticals at selected areas by integrating therapeutic compounds inside electrospun nanofibers, allowing controlled and localized drug delivery. The porous nature of electrospun MIP nanofibers allows for effective drug diffusion and promotes therapeutic efficacy [57].

Electrospinning has also enabled the development of MIP-based membranes for enhanced separation and purification operations. These membranes, which are made of electrospun MIP nanofibers, have high selectivity and permeability, allowing for the effective removal of particular pollutants from complicated combinations. Membranes of this type have found use in environmental cleanup, water treatment, and pharmaceutical purification operations [24,58]. While the combination of electrospinning and MIPs has shown enormous promise, problems persist. The optimization of MIP synthesis processes and electrospinning parameters, as well as production scalability, are active research topics. Efforts are also being made to increase the stability and reusability of MIP-infused electrospun nanofibers for long-term and efficient usage.

Electrospinning combined with MIPs has resulted in a new age of powerful molecular recognition systems. The unique features of electrospun nanofibers, along with MIPs’ specific binding capabilities, provide enormous promise for a wide range of applications, including biosensors, drug delivery, and separation procedures. As researchers continue to investigate and enhance this synergistic combination, we may anticipate additional breakthroughs in the sector. Table 1 provides a summary of recent studies that utilize electrospun nanofibers combined with MIPs for the removal of various pollutants from water streams [26,30,59,60,61,62,63,64,65,66].

## 2. Remediations

### 2.1. Dyes

Dyes are substances used to impart color to various materials, including textiles, paper, plastics, and inks. Their widespread use has led to environmental concerns due to their potential to contaminate water bodies. To address this issue, the occurrence, quantification, detection, and removal of dyes in water have been topics of research and development. Metal–organic frameworks (MOFs) with immobilized metal species (MIMs) have emerged as potential materials for dye removal [67].

#### 2.1.1. Occurrence and Quantification of Dyes in Water

Dyes can enter water bodies through various sources, including industrial effluent, textile dyeing processes, and runoff from agricultural fields. Their presence in water can have detrimental effects on aquatic ecosystems, including reduced light penetration, oxygen depletion, and toxicity to aquatic organisms [68]. The concentration of dyes in water must be quantified in order to assess water quality and apply remedial techniques. Quantification techniques that are often used include spectrophotometry, high-performance liquid chromatography (HPLC), and colorimetric approaches. To quantify the quantity of dye molecules in water samples, these approaches rely on assessing their absorption or emission properties. Detecting dyes in water is crucial for identifying contaminated sources and monitoring the efficiency of dye removal processes. Several techniques are used for the detection of dyes, including: UV–Vis Spectroscopy, fluorescence spectroscopy, colorimetry, etc. [69].

#### 2.1.2. Electrospun MIMs and Dye Removal Techniques

To remove dyes from water, several approaches have been devised, ranging from physical procedures to complex chemical processes. Among the most prevalent approaches are coagulation, adsorption, membrane separation and advanced oxidation processes. In the case of the coagulation process, chemical coagulants are added to the water, causing the dye particles to agglomerate and become easier to remove by sedimentation or filtering [70]. On the other hand, the adsorption technique involves dye molecules binding to the surface of a solid substance. Adsorbents for dye removal that are often utilized include activated carbon, zeolites, and clay minerals [71,72]. Yet membrane technologies, including reverse osmosis, nanofiltration, and ultrafiltration, may successfully remove dyes from water by physically separating them via a semipermeable substrate [73]. Advanced oxidation processes (AOPs) for the elimination of dyes generate highly reactive oxidizing species such as hydroxyl radicals. AOPs incorporate techniques such as ozone oxidation, photocatalysis, and Fenton’s reagent [74].

**Figure 2:**
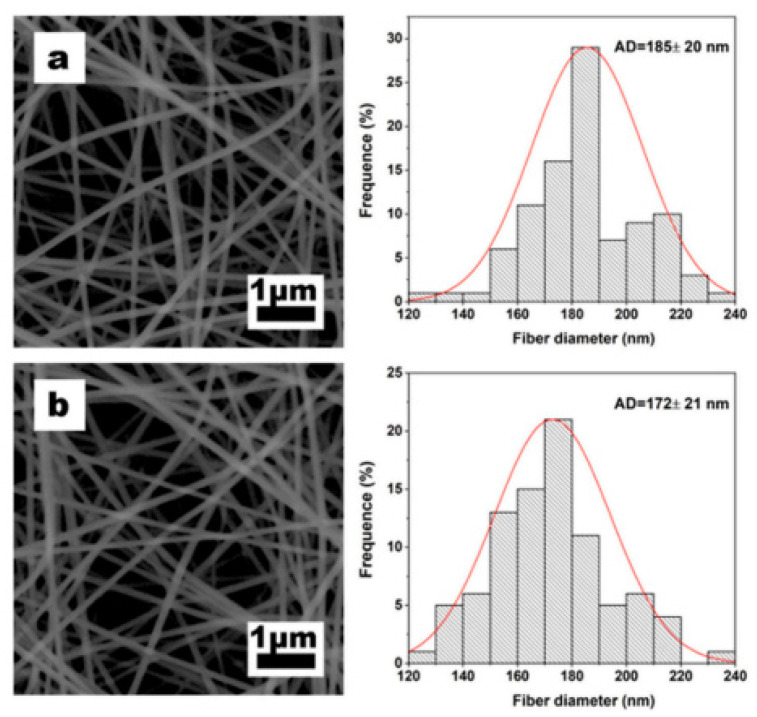
Electrospun nanofibrous membrane SEM images and diameter distributions (**a**) SA/PEO-NINM, and (**b**) SA/PEO-MINM [25].

Electrospun MIMs (molecularly imprinted membranes) have demonstrated promise in the removal of dyes from water systems. Dye-contaminated wastewater from sectors such as textile production may be removed more successfully, avoiding dyes from being discharged into natural water bodies. This contributes to the protection of aquatic habitats and the preservation of water quality. A study by Gao et al. [25] discovered that the inclusion of the template molecule methylene blue (MB) had no effect on the creation of nanofibers. Furthermore, the shape of the resulting nanofibers was not disrupted by the molecular imprinting process, indicating that the electrospun nanofibrous membranes were extremely stable. The nanofibrous membranes were appealing because of their intrinsic porous nature, with pore sizes varying from tens to hundreds of nanometers, allowing dye penetration and adsorption. Figure 2 is a representation of the SEM image showing the morphology of the resultant nanofibers. Electrospun nanofibrous membrane SEM images and diameter distributions: (a) sodium alginate/polyethylene oxide non-imprinted nanofibrous membrane (SA/PEO-NINM), (b) sodium alginate/polyethylene oxide molecularly imprinted nanofibrous membrane SA/PEO-(MINM).

**Figure 3:**
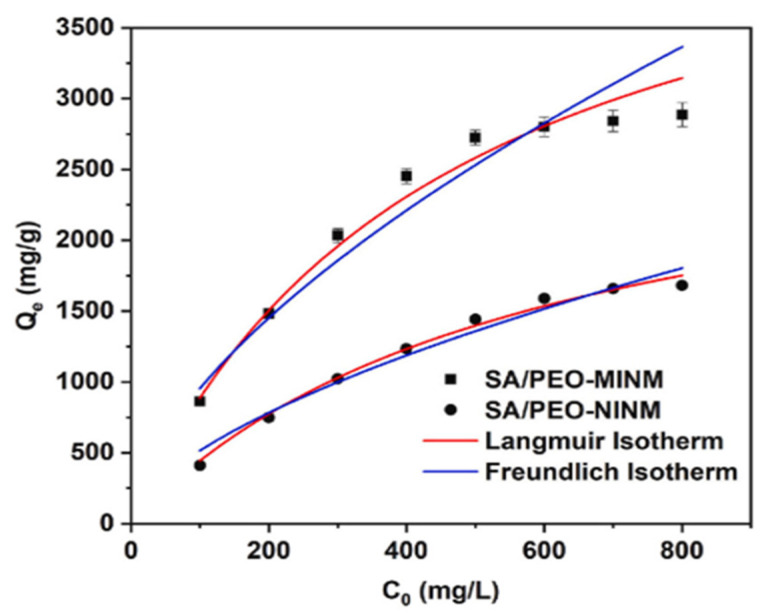
Adsorption isotherms of MB on the SA/PEO-NINM and SA/PEO-MINM [25].

As shown in Figure 3 and Table 2, the adsorption isotherms followed the Langmuir isotherm model. The two nanofibrous membranes functioned well in adsorption, with the initial MB concentrations ranging from 100 to 800 mg/L. During the adsorption process, the high starting concentration of MB solution increased MB adsorption by the nanofibrous membranes under the impact of the concentration gradient. Furthermore, the surface of SA/PEO-MINM contained more adsorption sites than the surface of SA/PEO-NINM. The adsorption thermodynamics of the two nanofibrous membranes were investigated using the Langmuir isotherm (Equation (1)) and Freundlich isotherm (Equation (2)) models.

(1) $$\frac{C_{e}}{Q_{e}}=\frac{C_{e}}{Q_{m}}+\frac{1}{Q_{m}b}\;$$

(2) $$\log Q_{e}=\log K_{f}+\frac{1}{n}\log C_{e}$$

The Langmuir isotherm model was adequate for clarifying the adsorption process; the value of R^2^ on SA/PEO-MINM was 0.997, which was significantly higher than the value of 0.990 on SA/PEO-NINM, Figure 2b. Methylene blue adsorption on SA/PEO-MINM was found to be monolayer adsorption with homogenous adsorption site distribution. Meanwhile, the SA/PEO-MINM demonstrated good adsorption capability, demonstrating the effective formation of imprinted cavities on the nanofibrous membrane.

**Figure 4:**
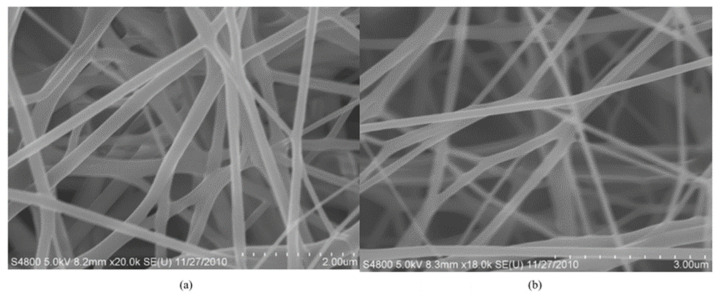
SEM images of electrospinning MIMs (**a**) and NIMs (**b**) [65].

**Figure 5:**
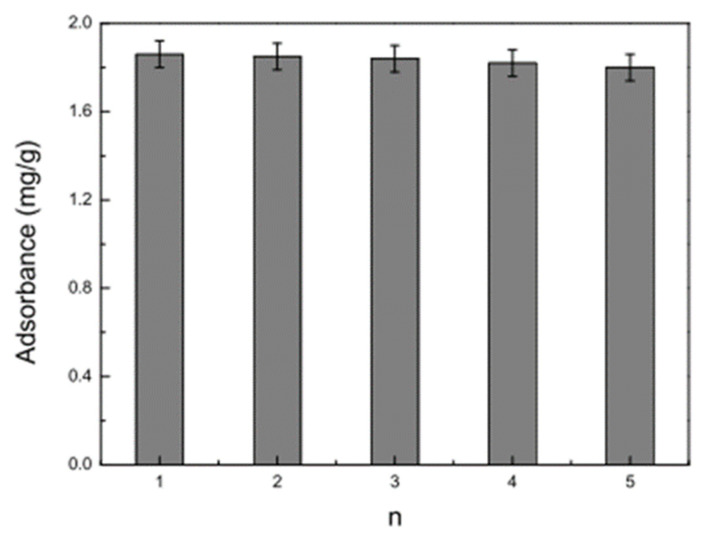
Absorbance change between different MIMs for RhB application [65].

According to SEM analysis, in a study Li et al. [65], the MIP wire and blank non-imprinted polymers (NIP) exhibited smooth surfaces and good morphology. The long electrospun nanofibers were scattered randomly and had remarkably homogeneous and dense structures. The nanofibers had an acceptable shape, with an average fiber diameter of 100–200 nm, as shown in Figure 3. The addition of the RhB template molecule to the initial polymer solution had no discernible effect on the morphology of the nanofibers. The electrospinning process began with a homogenous polymer solution comprising PET and RhB template molecularly imprinted microspheres. MIMs’ stability and repeatability were investigated. The adsorption of MIMs for RhB was not considerably reduced after eluting multiple times (n > 5) with pure methanol, as illustrated in Figure 4 and Figure 5. As a result, the findings indicate that MIMs have strong regeneration adsorption and availability [65].

**Figure 6:**
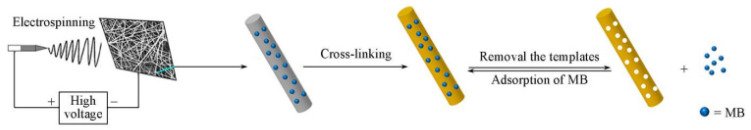
Schematic representation of synthesis of imprinted Sericin/PVA electrospun nanofibers. Reprinted with permission from [26].

Zhao et al. [26] developed molecularly imprinted sericin/PVA nanofibers for the selective removal of methylene blue (MB). To prevent PVA from dissolving during adsorption studies, the authors first crosslinked the nanofibers using glutaraldehyde vapor, followed by washing with hydrochloric acid (HCl) to effectively remove MB from the fibers, as illustrated in Figure 6. The resulting imprinted fibers demonstrated good stability, maintaining their nanofibrous structure after soaking in water for 48 h across a pH range of 3 to 11. The tensile strength and elongation-at-break of the imprinted, crosslinked nanofibers were approximately 8.2 MPa and 22.9%, respectively. The optimal adsorption occurred at pH 7, with an adsorption capacity of about 223 mg/g, significantly higher than the 108 mg/g observed for the non-imprinted sample. The selectivity ratios of the imprinted membrane for MB over CR, MO, and RhB were 6.2, 8.2, and 54.4, respectively. Additionally, the membrane retained approximately 94% of its initial adsorption capacity after five adsorption–regeneration cycles.

### 2.2. Heavy Metals

Heavy metals are elements that exist naturally and have large atomic weights and densities. When present in high quantities in water, they can be hazardous to human health and the environment. Concerning heavy metals include lead, mercury, cadmium, arsenic, chromium, and copper [75,76]. Heavy metals in water can be linked to natural causes, such as rock weathering and soil erosion, as well as anthropogenic activities, such as industrial operations, mining, and inappropriate waste disposal [77]. South Africa, like many other nations, suffers heavy metal pollution issues in its water sources. The prevalence of heavy metals in the aquatic environment has been exacerbated by the country’s industrial activity, mining operations, and urbanization processes. The presence of heavy metals in South African seas has prompted concerns about possible threats to human health and ecosystems. Heavy metals such as lead, mercury, cadmium, and arsenic have been found in numerous water sources around the country, according to studies [78].

#### 2.2.1. Occurrence and Quantification of Heavy Metals in Water

Heavy metals, once deposited into water, can remain and build over time, posing dangers to ecosystems and human populations. Heavy metal measurement in water is critical for evaluating the level of pollution and effective remediation procedures. Heavy metal identification and quantification are accomplished using a variety of approaches. After these heavy metals have been detected and quantified, several removal techniques are used.

One such technique is precipitation, whereby chemicals like lime, ferric chloride, or alum are added to water to generate insoluble precipitates containing heavy metal ions, which may subsequently be removed using sedimentation or filtering [79]. Ion exchange is another technique in which heavy metal ions in water are exchanged for ions of comparable charge but lesser toxicity. Ion exchange media such as zeolites and activated carbon can be employed [80,81]. When it comes to adsorption through chemical or physical interactions, activated carbon, activated alumina, and other adsorbent materials can be used to attract and trap heavy metal ions from water. Reverse osmosis (RO) is another typical adsorption technique where a membrane-based filtering process employs pressure to drive water through a semipermeable membrane, successfully eliminating heavy metal ions as well as other pollutants [82].

#### 2.2.2. Electrospun MIMs and Heavy Metal Removal Techniques

**Figure 7:**
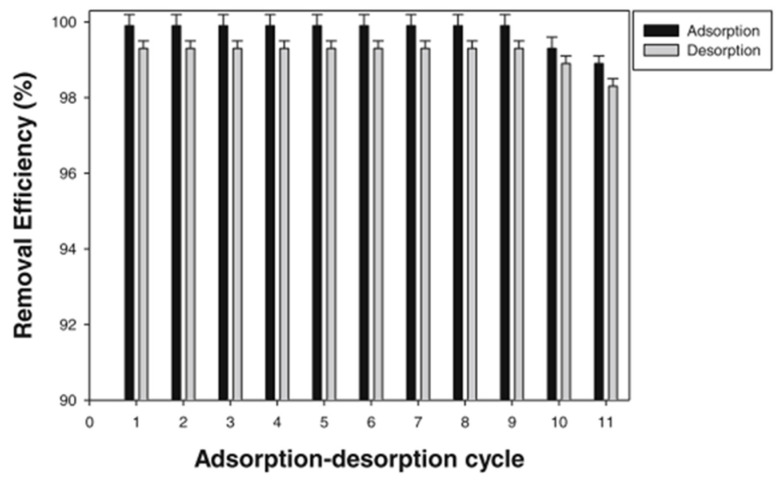
Amounts of NTPP adsorbed by the MIN and subsequently desorbed amounts in eleven adsorption desorption cycles.

In terms of the effect of molecularly imprinted membranes (MIMs) on heavy metal removal in water systems, MIMs have shown promise in heavy metal removal due to their high binding capacity, selectivity, and stability. They are designed to attach to certain heavy metal ions, allowing for effective removal from water. MIMs can be employed in filtration systems, membranes, or even as beads or resins for batch-wise water treatment [30]. Furthermore, MIMs have strong binding capabilities, allowing for efficient heavy metal removal even at low concentrations [83]. MIMs’ influence on heavy metal removal in water systems has been established in the scientific literature. MIMs have been shown in experiments to be successful at selectively removing heavy metals such as lead, mercury, and cadmium from polluted water sources. MIMs can be employed in filtration systems, membranes, or as beads or resins in batch water treatment [83]. The fabrication of molecularly imprinted fibers using an electrospinning process is a very promising approach that might be employed in foundational research of molecularly imprinted sorbent materials since it eliminates the requirement to grind bulk polymeric materials, which results in surfaces with nonspecific binding. In a study conducted by Awokoye et al. [84], they investigated two crucial characteristics of any sorbent material, in addition to selectivity and sensitivity: stability and reusability. Studies were done to investigate whether the nickel- 5,10,15,20-tetraphenylporphine (NTPP) bound to the molecularly imprinted nanofibers (MIN) could be desorbed/released and used in a new experiment. The desorption ratio was discovered to be quite high at 99.3%. To determine the molecularly imprinted nanocomposites’ (MINs’) reusability, the adsorption–desorption cycle was done 11 times using the imprinted material. Figure 7 demonstrates that the imprinted fiber was stable for up to nine binding/regeneration cycles with no decrease in NTPP removal efficiency and that the amount of NTPP bound by the MIN decreased in the tenth cycle. These findings suggested that the NTPP-MIN might be used repeatedly with no substantial reduction in removal efficiency.

Liu et al. [59] studied the use of Cu(II)-imprinted electrospun nanofibers for the removal of copper from wastewater streams. The nanofibers produced were uniform, with an average diameter of 290 nm; however, after washing and drying to remove the template, the diameter increased to approximately 490 nm. The adsorption capacity of the imprinted nanofibrous membrane was observed to increase with rising temperature. The membrane exhibited selectivity coefficients of approximately 52, 54, and 66 for Cu(II) compared to Pb(II), Ni(II), and Zn(II), respectively. Regeneration tests over five cycles showed that the membrane retained about 88% of its initial adsorption capacity. This suggests that these types of membranes can be utilized to recover or remove various targeted metalloids from water streams. In another study, hollow carbon nanofibers decorated with MnO_2_ nanosheets were developed for targeting lead (Pb) [60]. Polyacrylonitrile (PAN) was electrospun, followed by oxidation and carbonization. The carbonized fibers were then immersed in a KMnO_4_ solution and subjected to a hydrothermal process to produce hollow carbon nanofibers coated with manganese oxide nanosheets. The membrane exhibited a maximum adsorption capacity of approximately 461 mg/g. It also demonstrated excellent recyclability and removal efficiency, retaining about 93% of its initial adsorption capacity after five adsorption–desorption cycles. Li et al. [63] reported that a maximum adsorption capacity of 577 mg/g at pH 6 can be achieved using chitosan-imprinted fibers. The high adsorption was attributed to the large specific surface area resulting from finer nanofibers, the combined effects of physical chelation and chemical adsorption due to the presence of hydroxyl (OH) and amine (NH_2_) groups, as well as specific recognition sites created by the imprinting template. The imprinted membrane showed a preference for lead over other metalloids, although it also exhibited adsorption capacities of around 300 mg/g for Cu(II), Cd(II), and Ni(II), which is attributed to the membrane’s high surface area and porous structure. However, a notable limitation of the imprinted membrane is its lack of reusability. The adsorption capacity decreased to 359 mg/g after the second adsorption–desorption cycle and further dropped to 121 mg/g by the third cycle. This suggests that although chemical adsorption via available functional groups occurs, it hinders the effective removal of adsorbed metal ions, making the adsorbent suitable mainly for single-use applications. Such a limitation could lead to increased costs when treating large volumes of wastewater.

### 2.3. Emerging Pollutants (NSAIDs and ARVs)

Antiretroviral drugs (ARVs) and non-steroidal anti-inflammatory drugs (NSAIDs) are pharmacological substances that can infiltrate water systems via a variety of pathways. The most common sources of introduction include human and animal excretion, erroneous disposal of unwanted drugs, and insufficient removal during wastewater treatment operations [85]. Wastewater treatment plants (WWTPs) are critical in eliminating pollutants from wastewater, including pharmaceutical substances. Conventional treatment techniques, on the other hand, may not adequately eradicate all pharmaceutical residues, resulting in their presence in treated effluents. These effluents are often released into rivers, lakes, and other bodies of water, potentially contaminating aquatic habitats [1].

Various analytical approaches are used to measure the amounts of NSAIDs and ARVs in water systems. High-performance liquid chromatography (HPLC), liquid chromatography–mass spectrometry (LC-MS), gas chromatography–mass spectrometry (GC-MS), and enzyme-linked immunosorbent assay (ELISA) are examples of these procedures. These methods allow for the identification and quantification of pharmaceutical compounds in low quantities.

#### 2.3.1. Occurrence and Quantification of NSAIDs and ARVs in Water

When individuals take NSAIDs or ARVs, their bodies metabolize the drugs and some of the chemicals are expelled in their urine and feces. Pharmaceutical residues can then enter the sewage system via toilets and other sanitation facilities. Furthermore, drugs can enter water systems directly if they are improperly discarded by flushing them down the toilet or pouring them into sinks [86]. NSAIDs and ARVs are removed from water systems using a variety of methods. These approaches may be divided into three categories: physical, chemical, and biological processes. Activated carbon adsorption, membrane filtration, and advanced oxidation processes (AOPs) are examples of physical approaches. To break down pharmaceuticals into less hazardous compounds, chemical procedures are utilized, such as oxidation, reduction, or hydrolysis processes. Microorganisms or enzymes are used in biological processes to biodegrade pharmaceutical compounds [1].

#### 2.3.2. Removal Techniques for NSAIDs and ARVs

Solid-phase extraction is a popular technique for extracting and purifying diverse chemicals from complicated matrices, such as water samples. In the case of pharmaceuticals, such as NSAIDs and ARVs, SPE can be used to remove them from water systems. Samples are typically prepared by filtering and pH correction to assure compatibility with the SPE technique. The selection of the proper SPE sorbent is critical for successful removal [87]. Adsorbents that are often employed for pharmaceutical elimination include activated carbon, silica gel, and polymeric resins. These sorbents have an affinity for the target pharmaceuticals, allowing them to be absorbed from water [1]. SPE has a number of benefits, including excellent selectivity, adaptability, and the capacity to handle huge sample quantities [88]. It should be emphasized, however, that SPE is a sample-specific technique, and the selection of sorbent as well as procedure optimization are critical for effective pharmaceutical removal.

**Figure 8:**
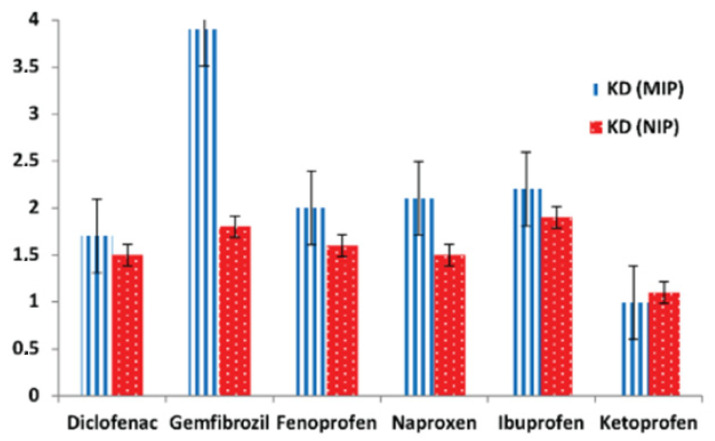
Selectivity diagram of the multi-template in the presences of a competitor ketoprofen [3].

The removal of NSAIDs and ARVs from water systems has shown potential using MIPs. However, the treatment of South African water may necessitate more investigation and evaluation. Several studies have shown that MIPs may successfully remove pharmaceuticals such as NSAIDs and ARVs from water systems in various regions [3,89,90]. Comparative studies can be carried out to evaluate the performance of MIPs in comparison to other removal procedures used in South Africa. While MIPs have shown success in removing NSAIDs and ARVs from water systems, their specific efficacy in global waters has to be investigated further. For example, in a study by Sibusiso et al. [3] where they synthesized a multi-template molecularly imprinted polymer for the removal of NSAIDs, they found that, because of the advantages of employing multiple templates, MIP obtained higher extraction efficiency for all target compounds than NIP. The adsorption kinetics were best suited with pseudo-second-order, indicating chemisorption. Despite parallels in polymer characterization results for MIP and NIP, experiments on selectivity for MIP demonstrated a high selectivity towards one compound (gemfibrozil) than other compounds. A Selectivity study diagram of templates in the presence of competitor for this study is shown in Figure 8. The MIP demonstrated significant adsorption efficiencies for the five targeted pharmaceuticals in the presence of two competitors, according to the data shown in Figure 7. The order of the KD values on MIP selectivity was gemfibrozil > ibuprofen > naproxen > fenoprofen > diclofenac, which could imply that the imprinting cavities of the compounds were created based on the interaction of shape, size, amount of hydrogen bonding, and functionality of the template. Some compounds are favored over others even in multi-template MIPs.

## 3. Other Pollutants Removal Using MIMs

A wide variety of pesticides, including paraoxon, parathion, and malathion, have been extensively used in agriculture [91]. These pesticides are well-known for their toxicity, carcinogenic potential, and tendency to bioaccumulate, prompting the development of various removal and degradation technologies such as chemical degradation, biological oxidation, and physical adsorption. Recently, there has been growing interest in employing electrospun molecularly imprinted nanofibers for the effective removal and/or degradation of these harmful pollutants. In this context, molecularly imprinted nanoparticles, synthesized using p-nitrophenol as the template via mini-emulsion polymerization, were incorporated into polyacrylonitrile (PAN) as the electrospinning vehicle to produce molecularly imprinted nanofibers [91]. The distribution of these nanoparticles on the surface of the nanofibers depended on the amount of imprinted nanoparticles introduced into the PET spinning solution. At nanoparticle loadings below 50 wt%, the fibers appeared smoother, indicating that the nanoparticles were mostly encapsulated within the polymer matrix. The resulting imprinted nanofibers demonstrated enhanced adsorption affinity toward p-nitrophenol. Moreover, the hydrolysis rate of paraoxon using these imprinted nanofibers was found to be 3.7 times higher compared to non-imprinted controls. Another group of concerning pollutants are phenolic compounds, such as bisphenol A (BPA), which are commonly released from everyday products [92,93,94]. Liu et al. [94] developed BPA-imprinted polymeric nanoparticles encapsulated within polyvinyl alcohol (PVA) electrospun nanofibers via electrospinning. These nanofibers were further functionalized by immobilizing *Pseudomonas aeruginosa* bacteria to facilitate the biodegradation of BPA. The imprinted nanofibers demonstrated a high adsorption capacity for BPA on their surface, thereby enhancing the accessibility of the pollutant to the bacteria for effective degradation. Moreover, the biodegradation efficiency increased in the presence of heavy metal ions, humic acid, as well as in surface water and wastewater treatment conditions. In a continuous flow system, the imprinted nanofibers achieved sustained BPA removal, highlighting their potential as a practical and efficient technology for removing and degrading various pollutants from water.

Gore et al. [95] investigated the use of ion-imprinted electrospun nanofibers for the removal of radioactive waste. The authors electrospun chitosan/PVA blended with 1-butyl-3-methylimidazolium tetrafluoroborate, producing uniform fibers with an average diameter of approximately 30 nm for removal of thorium (IV) ions. Adsorption was found to increase as pH rose, reaching a peak at neutral pH, after which it slightly declined. This behavior was attributed to the release of H^+^ ions into the solution, which reduced interactions with the target Th(IV) ions. The imprinted membrane achieved a maximum adsorption efficiency of around 90% at pH 7 and 25 °C within 2 h. Moreover, the membrane maintained adsorption capacity above 90% within 30 min over seven adsorption–desorption cycles. It is noteworthy that combining natural and synthetic polymers helps lower the cost of the resulting imprinted membrane; however, the vapor-phase crosslinking required can offset these savings by increasing the price to levels comparable to using either synthetic or natural polymers alone. Exploring other hydrophobic polymers combined with different components may further enhance the performance of electrospun imprinted materials.

## 4. Limitations

Polyvinyl alcohol (PVA) is currently the most widely utilized polymer matrix for encapsulating molecularly imprinted polymer (MIP) nanoparticles via electrospinning due to its water solubility and ease of processing. However, a fundamental limitation with PVA is the mandatory crosslinking step required to achieve fiber stability and water resistance. This crosslinking not only adds complexity but also elevates the production cost of the final MIP materials, potentially hindering large-scale and economically feasible applications. Therefore, the exploration of alternative biocompatible polymers that inherently possess sufficient stability without crosslinking is critically needed to reduce manufacturing costs and simplify processing.

Another key challenge lies in the predominance of traditional single-needle electrospinning techniques in the literature (see Table 1). While these methods provide proof-of-concept at the laboratory scale, their low throughput and poor scalability present significant barriers to commercial adoption. The field lacks comprehensive studies adopting advanced electrospinning techniques, such as needleless or multi-jet electrospinning, which promise higher productivity and process scalability. This gap underscores the urgent need for research efforts to optimize and validate scalable electrospinning approaches tailored for MIP nanoparticle immobilization.

Moreover, the spatial distribution of MIP nanoparticles within the nanofiber matrix remains suboptimal for maximum functionality. Conventional electrospinning typically results in nanoparticles being embedded inside the fiber bulk, limiting their accessibility for pollutant binding and degradation. Emerging methods, such as coaxial electrospinning, provide an attractive alternative by enabling predominant localization of MIP nanoparticles on the fiber surface, thereby enhancing interaction with target molecules. However, these advanced techniques have been underexplored, and systematic studies are required to establish effective fabrication protocols.

A further limitation is the insufficient reporting of electrospinning process parameters, especially solution properties and ambient conditions, in many published works. These parameters critically influence fiber morphology and MIP efficacy, yet detailed optimization data are rarely disclosed, impeding reproducibility and hindering meaningful comparisons across studies.

Finally, while electrospinning facilitates convenient recovery and reuse of MIP nanofiber materials, addressing the abovementioned limitations through interdisciplinary research and transparent reporting will be pivotal to unlocking the full potential of electrospun MIPs for practical, scalable, and cost-effective pollutant removal and degradation.

## 5. Future Outlook and Conclusions

Electrospun molecularly imprinted membranes present a promising alternative for the effective removal of variety of pollutants from water sources, including non-steroidal anti-inflammatory drugs (NSAIDs) and antiretrovirals (ARVs). Electrospinning is a versatile process for producing ultrafine fibers with sizes ranging from nano- to micrometers. Electrospinning, when paired with molecular imprinting technology, allows the generation of molecularly imprinted membranes (MIMs) with unique recognition sites for target molecules. Because of their customized molecular structure, MIMs are designed to selectively attach to and capture certain substances, such as pharmaceuticals. Pharmaceuticals, particularly NSAIDs and ARVs, provide a substantial challenge to water treatment systems due to their persistence and possible detrimental effects on human health and the environment. Conventional water treatment technologies frequently fail to remove these drugs successfully, prompting the exploration of new alternatives. In the removal of pharmaceuticals from water, electrospun molecularly imprinted membranes have various benefits. For instance, their high surface area-to-volume ratio and porous shape allow for more interaction between target molecules and recognition sites, improving overall binding efficiency. Furthermore, the molecular imprinting technique allows MIMs to be customized to preferentially target certain pharmaceutical molecules, improving selectivity and removal efficacy. Current studies on the use of electrospun MIMs in water treatment applications for dyes and heavy metal elimination have yielded encouraging results. These membranes have shown strong adsorption capabilities, great chemical stability, and acceptable reusability, making them an attractive choice for removing NSAIDs and ARVs from polluted water sources. Electrospun molecularly imprinted membranes have a lot of promise as a solution for removing drugs like NSAIDs and ARVs from water sources. They are an appealing solution for addressing the issues posed by pharmaceutical contamination in aquatic settings due to their unique combinations of high surface area, selectivity, and customizability. More research and development in this area can help to enhance efficient and sustainable water treatment methods. MIPs targeting particular substances in different water sources should be developed as part of research activities. MIPs’ performance in South African water matrices should be evaluated in terms of adsorption capacity, selectivity, regeneration capability, and long-term stability.

## Figures and Tables

**Table 1:** Summary of recent studies on electrospun MIPS for pollutants removal.

Formulation	Electrospinning Technique	Optimal Processing Conditions	Highlights	Refs.
Polyethylene terephthalate (PET)		-Voltage = 18 kV -Tip-to-collector (TTC) = 15 cm -RH = 45% -Temperature = 25 °C	An optimal pH of 7 and an adsorption time of 80 min were identified for RhB uptake. The system achieved over 90% recovery across five consecutive cycles.	[65]
Polyvinyl alcohol (PVA)/sercin-methylene blue (MB)	Classic electrospinning	-Voltage = 15 kV -Tip-to-collector (TTC) = 18 cm	The imprinted membrane was prepared by crosslinking with glutaraldehyde, followed by washing to remove the target molecule. The imprinted electrospun membrane demonstrated enhanced selectivity and superior adsorption capacity compared to the non-imprinted counterpart.	[26]
Polyvinyl alcohol (PVA)/Cu-L-histidine	Classic electrospinning	-Voltage = 26 kV -Feeding rate = 0.4 mL/h -TTC = 7 cm	After crosslinking using glutaraldehyde, the imprinted nanofibrous membrane was washed to remove the copper. The desorption capacity reached approximately 115 mg/g, maintaining about 88% of the initial adsorption capacity after five regeneration cycles. Selectivity coefficient of 52, 54 and 66 for Cu relative to Pb, Ni and Zn were attained.	[59]
Polyacrylonitrile (PAN)	Classic electrospinning	-	Following pre-oxidation and carbonization processes to convert PAN into carbon nanofibers, the fibers were immersed in a KMnO_4_ solution and subjected to a hydrothermal treatment to produce a hollow-structured membrane coated with manganese oxide nanosheets. The imprinted membrane exhibited its highest adsorption capacity at pH 6, reaching an optimal value of approximately 461 mg/g. After five adsorption–desorption cycles, the membrane retained about 81% of its initial adsorption capacity.	[60]
polysulphone (PSU)/nickel (II)-dimethyl glyoxime	Classic electrospinning	-Voltage = 15 kV -TTC = 12 cm	The imprinted membrane was employed for solid-phase extraction (SPE) and removal of nickel (Ni), achieving recovery rates above 90% even in the presence of interfering metal ions	[61]
Chitosan	Classic electrospinning	-Voltage = 18.5 kV -TTC = 9 cm	Chitosan combined with lead chloride was prepared via electrospinning, followed by crosslinking using glutaraldehyde vapor and washing with EDTA to remove the template. The imprinted fibers demonstrated an adsorption capacity of 577 mg/g at pH 6.	[63]
poly(ethyleneterephthalate) (PET)/propranolol		-Voltage = 20 kV -TTC = 20 cm	The resulting composite membrane exhibited a strong affinity for the target, effectively preventing any leakage from the membrane.	[62]
PVA/gelatine		-Voltage = 15 kV -Feeding rate = 0.25 mL/h -RH = 55% -TTC = 12 cm	Fibers with an average diameter of 38 nm were produced, featuring spherical linkages (beads). However, no adsorption studies were conducted; the inclusion of the template was confirmed solely through XPS analysis.	[66]
PVA		-TTC = 15 cm -Voltage = 20 kV	The fibers were crosslinked using butanediol diglycidyl ether, followed by washing steps. The nanofibers demonstrated approximately 100% recovery after the adsorption–desorption cycle.	[64]

**Table 2:** Langmuir and Freundlich isotherm parameters for MB adsorption on nanofibrous membranes [25].

Sample	Langmuir Isotherm			Freundlich Isotherm		
	Q_max_ (mg/g)	B (L/mg)	R^2^	K_f_ (mg/g)	n	R^2^
SA/PEO-MINM	3186.74	2.11 × 10^−2^	0.997	384.13	2.90	0.945
SA/PEO-NINM	2551.02	3.40 × 10^−3^	0.990	39.05	1.65	0.969

## References

[1] Sigonya S., Mokhothu T.H., Mokhena T.C., Makhanya T.R. (2023). Mitigation of Non-Steroidal Anti-Inflammatory and Antiretroviral Drugs as Environmental Pollutants by Adsorption Using Nanomaterials as Viable Solution—A Critical Review. Appl. Sci..

[2] Mlunguza N.Y., Ncube S., Nokwethemba Mahlambi P., Chimuka L., Madikizela L.M. (2019). Adsorbents and removal strategies of non-steroidal anti-inflammatory drugs from contaminated water bodies. J. Environ. Chem. Eng..

[3] Nkosi S.M., Mahlambi P.N., Chimuka L. (2022). Synthesis, characterisation and optimisation of bulk molecularly imprinted polymers from nonsteroidal anti-inflammatory drugs. S. Afr. J. Chem..

[4] Mlunguza N.Y., Mdluli P.S., Zunngu S.S., Madikizela L.M., Tavengwa N.T., Chimuka L. (2018). Application of molecularly imprinted polymer designed for the selective extraction of ketoprofen from wastewater. Water SA.

[5] Fan J.P., Luo J.J., Zhang X.H., Zhen B., Dong C.Y., Li Y.C., Shen J., Cheng Y.T., Chen H.P. (2019). A novel electrospun Β-CD/CS/PVA nanofiber membrane for simultaneous and rapid removal of organic micropollutants and heavy metal ions from water. Chem. Eng. J..

[6] Van Tran T., Nguyen D.T.C., Le H.T.N., Vo D.V.N., Nanda S., Nguyen T.D. (2020). Optimization, equilibrium, adsorption behavior and role of surface functional groups on graphene oxide-based nanocomposite towards diclofenac drug. J. Environ. Sci..

[7] Larsson E., Al-Hamimi S., Jönsson J.Å. (2014). Behaviour of nonsteroidal anti-inflammatory drugs and eight of their metabolites during wastewater treatment studied by hollow fibre liquid phase microextraction and liquid chromatography mass spectrometry. Sci. Total Environ..

[8] Megahed S.H., Abdel-Halim M., El-shabrawy Y.I., Saad E.M., Hefnawy A., Handoussa H., Mizaikoff B., El Gohary N.A. (2025). Design, synthesis and medical prospects of electrospun molecularly imprinted fibers. Sci. Rep..

[9] Patel K.D., Kim H.W., Knowles J.C., Poma A. (2020). Molecularly Imprinted Polymers and Electrospinning: Manufacturing Convergence for Next-Level Applications. Adv. Funct. Mater..

[10] Crapnell R.D., Street R.J., Ferreira-Silva V., Down M.P., Peeters M., Banks C.E. (2021). Electrospun Nylon Fibers with Integrated Polypyrrole Molecularly Imprinted Polymers for the Detection of Glucose. Anal. Chem..

[11] Belbruno J.J. (2019). Molecularly Imprinted Polymers. Chem. Rev..

[12] Vasapollo G., Del Sole R., Mergola L., Lazzoi M.R., Scardino A., Scorrano S., Mele G. (2011). Molecularly imprinted polymers: Present and future prospective. Int. J. Mol. Sci..

[13] Boysen R.I. (2019). Advances in the development of molecularly imprinted polymers for the separation and analysis of proteins with liquid chromatography. J. Sep. Sci..

[14] Cheong W.J., Yang S.H., Ali F. (2013). Molecular imprinted polymers for separation science: A review of reviews. J. Sep. Sci..

[15] Fu J., Chen L., Li J., Zhang Z. (2015). Current status and challenges of ion imprinting. J. Mater. Chem. A.

[16] Zahedi P., Fallah-Darrehchi M., Nadoushan S.A., Aeinehvand R., Bagheri L., Najafi M. (2017). Morphological, thermal and drug release studies of poly (methacrylic acid)-based molecularly imprinted polymer nanoparticles immobilized in electrospun poly (ε-caprolactone) nanofibers as dexamethasone delivery system. Korean J. Chem. Eng..

[17] Piletsky S., Canfarotta F., Poma A., Bossi A.M., Piletsky S. (2020). Molecularly Imprinted Polymers for Cell Recognition. Trends Biotechnol..

[18] Yang X., Li X., Zhang L., Gong J. (2017). Electrospun template directed molecularly imprinted nanofibers incorporated with BiOI nanoflake arrays as photoactive electrode for photoelectrochemical detection of triphenyl phosphate. Biosens. Bioelectron..

[19] Cieplak M., Kutner W. (2016). Artificial Biosensors: How Can Molecular Imprinting Mimic Biorecognition?. Trends Biotechnol..

[20] Gao D., Wang D.D., Zhang Q., Yang F.Q., Xia Z.N., Zhang Q.H., Yuan C.S. (2017). In Vivo Selective Capture and Rapid Identification of Luteolin and Its Metabolites in Rat Livers by Molecularly Imprinted Solid-Phase Microextraction. J. Agric. Food Chem..

[21] Peng S., Wen Y., Ming Y., Huang T., Xu G., Yan J., Huang J., Song Z., Wang W., Breadmore M.C. (2026). Molecularly imprinted polymers based enrichment and separation for trace analysis in capillary electrophoresis. Talanta.

[22] Demirkurt M., Olcer Y.A., Demir M.M., Eroglu A.E. (2018). Electrospun polystyrene fibers knitted around imprinted acrylate microspheres as sorbent for paraben derivatives. Anal. Chim. Acta.

[23] Ali Z., Ahmad R. (2020). Nanotechnology for Water Treatment.

[24] Zhu F., Zheng Y.M., Zhang B.G., Dai Y.R. (2021). A critical review on the electrospun nanofibrous membranes for the adsorption of heavy metals in water treatment. J. Hazard. Mater..

[25] Gao T., Guan G., Wang X., Lou T. (2022). Electrospun molecularly imprinted sodium alginate/polyethylene oxide nanofibrous membranes for selective adsorption of methylene blue. Int. J. Biol. Macromol..

[26] Zhao R., Li X., Sun B., Li Y., Li Y., Wang C. (2017). Preparation of molecularly imprinted sericin/poly(vinyl alcohol) electrospun fibers for selective removal of methylene blue. Chem. Res. Chin. Univ..

[27] Kebede T.G., Dube S., Nindi M.M. (2019). Biopolymer electrospun nanofibres for the adsorption of pharmaceuticals from water systems. J. Environ. Chem. Eng..

[28] Camiré A., Espinasse J., Chabot B., Lajeunesse A. (2020). Development of electrospun lignin nanofibers for the adsorption of pharmaceutical contaminants in wastewater. Environ. Sci. Pollut. Res..

[29] Ostovan A., Arabi M., Wang Y., Li J., Li B., Wang X., Chen L. (2022). Greenificated Molecularly Imprinted Materials for Advanced Applications. Adv. Mater..

[30] Yoshikawa M., Tharpa K., Dima Ş.O. (2016). Molecularly Imprinted Membranes: Past, Present, and Future. Chem. Rev..

[31] Kempe M., Mosbach K. (1995). Separation of amino acids, peptides and proteins on molecularly imprinted stationary phases. J. Chromatogr. A.

[32] Erdo J. (2016). Trends in Analytical Chemistry Electrosynthesized molecularly imprinted polymers for protein recognition. TrAC Trends Anal. Chem..

[33] Janiak D.S., Kofinas P. (2007). Molecular imprinting of peptides and proteins in aqueous media. Anal. Bioanal. Chem..

[34] Yang H., Liu H.B., Tang Z.S., Qiu Z.D., Zhu H.X., Song Z.X., Jia A.L. (2021). Synthesis, performance, and application of molecularly imprinted membranes: A review. J. Environ. Chem. Eng..

[35] Kang M.S., Lee J.H., Kim K.S. (2025). Small Toxic Molecule Detection and Elimination Using Molecularly Imprinted Polymers (MIPs). Biosensors.

[36] Rashid S., Rohit J.V. (2025). Molecularly Imprinted Polymer Nanoparticles and Imprinted Nanocomposites Based Optical Sensors for the Detection of Chemical Pollutants. J. Inorg. Organomet. Polym. Mater..

[37] Chen W., Ma Y., Pan J., Meng Z., Pan G., Sellergren B. (2015). Molecularly imprinted polymers with stimuli-responsive affinity: Progress and perspectives. Polymers.

[38] Syed Yaacob S.F.F., Suwaibatu M., Raja Jamil R.Z., Mohamed Zain N.N., Raoov M., Mohd Suah F.B. (2023). Review of molecular imprinting polymer: Basic characteristics and removal of phenolic contaminants based on the functionalized cyclodextrin monomer. J. Chem. Technol. Biotechnol..

[39] Gao J., Chen L., Xing W., Yu C., Yan Y., Wu Y. (2023). “Nanomagnet-inspired” design on molecularly imprinted nanofiber membrane: Mechanisms for improved transport selectivity of sufficient specific sites. J. Membr. Sci..

[40] Ahmed Y.W., Loukanov A., Tsai H.C. (2024). State-of-the-Art Synthesis of Porous Polymer Materials and Their Several Fantastic Biomedical Applications: A Review. Adv. Healthc. Mater..

[41] Rahman M., Dip T.M., Nur M.G., Hossain M.H., Snow F., Hossain N.B., Mirabedini A., Quigley A., Padhye R., Houshyar S. (2025). Nanomaterial-Integrated 3D Biofabricated Structures for Advanced Biomedical Applications. Macromol. Mater. Eng..

[42] Du Y., Yu D.G., Yi T. (2023). Electrospun Nanofibers as Chemosensors for Detecting Environmental Pollutants: A Review. Chemosensors.

[43] Macagnano A., Molinari F.N., Papa P., Mancini T., Lupi S., D’Arco A., Taddei A.R., Serrecchia S., De Cesare F. (2024). Nanofibrous Conductive Sensor for Limonene: One-Step Synthesis via Electrospinning and Molecular Imprinting. Nanomaterials.

[44] Wang S., She Y., Hong S., Du X., Yan M., Wang Y., Qi Y., Wang M., Jiang W., Wang J. (2019). Dual-template imprinted polymers for class-selective solid-phase extraction of seventeen triazine herbicides and metabolites in agro-products. J. Hazard. Mater..

[45] Hasanah A.N., Safitri N., Zulfa A., Neli N., Rahayu D. (2021). Factors affecting preparation of molecularly imprinted polymer and methods on finding template-monomer interaction as the key of selective properties of the materials. Molecules.

[46] Chen H.W., Lin M.F. (2020). Characterization, biocompatibility, and optimization of electrospun SF/PCL/CS composite nanofibers. Polymers.

[47] Hou Y., Ofori E.A., Gbologah L., Xiong Y., Mensah-Darkwa K., Tawiah B., Fei B., Zhao X. (2025). Electrochemical Fiber Electrode Fabrication by Spinning: State-of-the-Art and Perspectives. ACS Electrochem..

[48] Yıldız Ü.Y., Hussain C.G., Keçili R., Hussain C.M. (2024). Green approaches for the preparation of molecularly imprinted polymers. Green Imprinted Materials from Design to Environmental and Food Applications.

[49] Haghi A.K., Akbari M. (2007). Trends in electrospinning of natural nanofibers. Phys. Status Solidi Appl. Mater. Sci..

[50] Rai M., Biswas J.K. (2018). Nanomaterials: Ecotoxicity, Safety, and Public Perception.

[51] Li D., Xia Y. (2004). Electrospinning of nanofibers: Reinventing the wheel?. Adv. Mater..

[52] Chua C. (2002). Review The Design of Scaffolds for Use in Tissue Engineering. Part II. Rapid Prototyping Techniques. Tissue Eng..

[53] Mckee M.G., Layman J.M., Cashion M.P., Long T.E. (2006). Supporting Online Material for Phospholipid Non-woven Electrospun Membranes. Science.

[54] Ramakrishna S., Jose R., Archana P.S., Nair A.S., Balamurugan R., Venugopal J., Teo W.E. (2010). Science and engineering of electrospun nanofibers for advances in clean energy, water filtration, and regenerative medicine. J. Mater. Sci..

[55] Laudenslager M.J., Scheffler R.H., Sigmund W.M. (2010). Electrospun materials for energy harvesting, conversion, and storage: A review. Pure Appl. Chem..

[56] Bhardwaj N., Kundu S.C. (2010). Electrospinning: A fascinating fi ber fabrication technique. Biotechnol. Adv..

[57] Yong C., Wang Z., Zhang X., Shi X., Ni Z., Fu H., Ding G.S., Fu Z.R., Yin H. (2014). The therapeutic effect of monocyte chemoattractant protein-1 delivered by an electrospun scaffold for hyperglycemia and nephrotic disorders. Int. J. Nanomed..

[58] Wang R., Liu Y., Li B., Hsiao B.S., Chu B. (2012). Electrospun nanofibrous membranes for high flux microfiltration. J. Membr. Sci..

[59] Liu X., Yang J.L., Tong L.Y., Zhang Q., Li X.W., Chen J.D. (2015). Preparation of Cu(II)-imprinted nanofibers from co-electrospinning PVA and imprinting complex. Chem. Res. Chin. Univ..

[60] Li W., Li Y., Liu J., Chao S., Yang T., Li L., Wang C., Li X. (2021). A Novel Hollow Carbon@MnO_2_ Electrospun Nanofiber Adsorbent for Efficient Removal of Pb^2+^ in Wastewater. Chem. Res. Chin. Univ..

[61] Rammika M., Darko G., Torto N. (2011). Incorporation of Ni(II)-dimethylglyoxime ion-imprinted polymer into electrospun polysulphone nanofibre for the determination of Ni(II) ions from aqueous samples. Water SA.

[62] Yoshimatsu K., Ye L., Lindberg J., Chronakis I.S. (2008). Selective molecular adsorption using electrospun nanofiber affinity membranes. Biosens. Bioelectron..

[63] Li Y., Qiu T., Xu X. (2013). Preparation of lead-ion imprinted crosslinked electro-spun chitosan nanofiber mats and application in lead ions removal from aqueous solutions. Eur. Polym. J..

[64] Piperno S., Tse Sum Bui B., Haupt K., Gheber L.A. (2011). Immobilization of molecularly imprinted polymer nanoparticles in electrospun poly(vinyl alcohol) nanofibers. Langmuir.

[65] Li L., Liu H., Lei X., Zhai Y. (2012). Electrospun Nanofiber Membranes Containing Molecularly Imprinted Polymer (MIP) for Rhodamine B (RhB). Adv. Chem. Eng. Sci..

[66] Alfikro I., Afrizal N., Jorena J., Saleh K., Satya O., Virgo F., Royani I. (2024). Incorporation of Fe(III)-IIPs (Ion Imprinted Polymers) and PVA/Gelatine Nanofiber using Electrospinning Method: A Report.

[67] Bagbi Y., Pandey A., Solanki P.R. (2019). Electrospun Nanofibrous Filtration Membranes for Heavy Metals and Dye Removal. Nanoscale Materials in Water Purification.

[68] Nguyen C.H., Juang R.S. (2019). Efficient removal of cationic dyes from water by a combined adsorption-photocatalysis process using platinum-doped titanate nanomaterials. J. Taiwan Inst. Chem. Eng..

[69] Wang L., Li L., Cao D. (2017). A BODIPY-based dye with red fluorescence in solid state and used as a fluorescent and colorimetric probe for highly selective detection of cyanide. Sens. Actuators B Chem..

[70] Ouakouak A., Abdelhamid M., Thouraya B., Chahinez H. (2021). Development of a Novel Adsorbent Prepared from Dredging Sediment for Effective Removal of Dye in Aqueous Solutions. Appl. Sci..

[71] Shahadat M. (2018). Regeneration performance of clay-based adsorbents for the removal of industrial dyes: A review. RSC Adv..

[72] Shojaei M., Esmaeili H. (2022). Ultrasonic-assisted synthesis of zeolite/activated carbon @ MnO 2 composite as a novel adsorbent for treatment of wastewater containing methylene blue and brilliant blue. Environ. Monit. Assess..

[73] Abid M.F., Zablouk M.A., Abid-alameer A.M. (2012). Experimental study of dye removal from industrial wastewater by membrane technologies of reverse osmosis and nanofiltration. Iran. J. Environ. Health Sci. Eng..

[74] Peramune D., Manatunga D.C., Dassanayake R.S., Premalal V., Liyanage R.N., Gunathilake C., Abidi N. (2022). Recent advances in biopolymer-based advanced oxidation processes for dye removal applications: A review. Environ. Res..

[75] Li Q.G., Liu G.H., Qi L., Wang H.C., Ye Z.F., Zhao Q.L. (2022). Heavy metal-contained wastewater in China: Discharge, management and treatment. Sci. Total Environ..

[76] Mahurpawar M. (2015). Effects of Heavy Metals on Human Healtheffects of Heavy Metals on Human Health. Int. J. Res. Granthaalayah.

[77] Njoku P., Bennard O., Akudinobi B. (2020). Potential health risk and levels of heavy metals in water resources of lead—Zinc mining communities of Abakaliki, southeast Nigeria. Appl. Water Sci..

[78] Madikizela L.M., Chimuka L., Ncube S. (2021). Metal pollution source apportionment in two important Rivers of Eastern Cape Province, South Africa: A case study of Bizana and Mthatha Rivers. Environ. Forensics.

[79] Esalah J., Husein M.M. (2008). Removal of Heavy Metals from Aqueous Solutions by Precipitation-Filtration Using Novel Organo- Phosphorus Ligands. Sep. Sci. Technol..

[80] Pepe F., De Gennaro B., Aprea P., Caputo D. (2013). Natural zeolites for heavy metals removal from aqueous solutions: Modeling of the fixed bed Ba^2+^/Na^+^ ion-exchange process using a mixed phillipsite/chabazite-rich tuff. Chem. Eng. J..

[81] Dong L., Wang Z., Gu P., Chen G., Jiang R. (2018). A new function of spent activated carbon in BAC process: Removing heavy metals by ion exchange mechanism. J. Hazard. Mater..

[82] Zhao F., Xiang H., Min X., Tang C. (2022). Recent advances in membrane filtration for heavy metal removal from wastewater: A mini review. J. Water Process Eng..

[83] Shu C., Chiew C., Gourich W., Pasbakhsh P., Eong P., Tey T., Pin C., Chan E. (2022). Life cycle assessment on alginate-based nanocomposite beads for the removal of lead (II) from aqueous solutions. J. Water Process Eng..

[84] Awokoya K.N., Moronkola B.A., Chigome S., Ondigo D.A., Tshentu Z., Torto N. (2013). Molecularly imprinted electrospun nanofibers for adsorption of nickel-5,10,15,20-tetraphenylporphine (NTPP) in organic media. J. Polym. Res..

[85] Sigonya S., Chibuzor S., Phumlani O., Mdluli S., Hendrica T. (2022). Method optimisation and application based on solid phase extraction of non steroidal anti-inflammatory drugs, antiretroviral drugs, and a lipid regulator from coastal areas of Durban, South Africa. SN Appl. Sci..

[86] Eggen T., Moeder M., Arukwe A. (2010). Municipal landfill leachates: A significant source for new and emerging pollutants. Sci. Total Environ..

[87] Aini W., Ibrahim W., Veni K., Marsin M. (2012). Novel sol–gel hybrid methyltrimethoxysilane–tetraethoxysilane as solid phase extraction sorbent for organophosphorus pesticides. J. Chromatogr. A.

[88] Andrade-Eiroa A., Canle M., Leroy-Cancellieri V., Cerdà V. (2016). Solid-phase extraction of organic compounds: A critical review. part ii. TrAC Trends Anal. Chem..

[89] Mbhele Z.E., Ncube S., Madikizela L.M. (2018). Synthesis of a molecularly imprinted polymer and its application in selective extraction of fenoprofen from wastewater. Environ. Sci. Pollut. Res..

[90] Lagha A. (2011). A Molecularly Imprinted Polymer for the Selective Solid-Phase Extraction of Ibuprofen from Urine Samples. Open Chem. Biomed. Methods J..

[91] Zhang L., Guo Y., Chi W.H., Shi H.G., Ren H.Q., Guo T.Y. (2014). Electrospun nanofibers containing p-nitrophenol imprinted nanoparticles for the hydrolysis of paraoxon. Chin. J. Polym. Sci. (Engl. Ed.).

[92] Chang K.L., Teng T.C., Fu C.K., Liu C.H. (2019). Improving biodegradation of Bisphenol A by immobilization and inducer. Process Saf. Environ. Prot..

[93] Wu Y.T., Zhang Y.H., Zhang M., Liu F., Wan Y.C., Huang Z., Ye L., Zhou Q., Shi Y., Lu B. (2014). Selective and simultaneous determination of trace bisphenol A and tebuconazole in vegetable and juice samples by membrane-based molecularly imprinted solid-phase extraction and HPLC. Food Chem..

[94] Liu F., Liu Q., Zhang Y., Liu Y., Wan Y., Gao K., Huang Y., Xia W., Wang H., Shi Y. (2015). Molecularly imprinted nanofiber membranes enhanced biodegradation of trace bisphenol A by Pseudomonas aeruginosa. Chem. Eng. J..

[95] Gore P.M., Khurana L., Siddique S., Panicker A., Kandasubramanian B. (2018). Ion-imprinted electrospun nanofibers of chitosan/1-butyl-3-methylimidazolium tetrafluoroborate for the dynamic expulsion of thorium (IV) ions from mimicked effluents. Environ. Sci. Pollut. Res..

